# Interstitiality in the smart city: More than top-down and bottom-up smartness

**DOI:** 10.1177/00420980221097590

**Published:** 2022-06-24

**Authors:** Ryan Burns, Preston Welker

**Affiliations:** University of Calgary, Canada; University of Calgary, Canada

**Keywords:** community, digital geographies, inequality, politics, smart cities, technology

## Abstract

The critical research agenda on smart cities has tended to assume a largely top-down orientation in which powerful actors like the state and corporations enact programmes to embed Information & Communication Technologies (ICT) in the urban landscape. Because of the way research has framed this relation of power, the dominant response has been to seek social justice by either contesting these top-down exercises of (digital) power or by reconceptualising the smart city ‘from below’. In this paper, we join a growing chorus of voices recognising the importance of interstitial actors that influence the ways in which the smart city manifests. We draw on a five-year ongoing study in Calgary, Alberta, to examine two actor groups that are, properly, neither top-down nor bottom-up, but play an important role in envisioning, implementing and contesting how ‘smartness’ is framed. The first set of actors, situated between the top and bottom of the smart city hierarchy, are most prominently community associations, non-profit organisations and ad-hoc task groups. The second group is comprised of groups with different digital practices, whose spectre of marginalisation influences how digital systems are articulated and pursued. These actors strategically move between different interstices in order to enact particular kinds of political influence, and often influence smart cities by virtue of their absence, profoundly impacting urban political geographies of smartness.

## 摘要

智慧城市的关键研究议程倾向于采用自上而下的方向，其中，政府和企业等强大的参与者制定计划，将信息和通信技术 (ICT) 嵌入城市景观。由于研究对这种权力关系的构建方式，主要的反应是通过对这些自上而下的（数字）权力行使的挑战、或通过“自下而上”重新概念化智慧城市来寻求社会正义。在本文中，我们代表一种越来越广泛的声音，即主张影响智慧城市表现方式的间隙行为者的重要性。我们借鉴了当前在阿尔伯塔省卡尔加里市进行的一项为期五年的研究，以检验两个行为者群体，它们既不是纯粹的自上而下也不是纯粹的自下而上，但在构想、实施和质疑“智慧”框架构建方面发挥着重要作用。第一组行为者位于智慧城市层级结构的顶部和底部之间，最突出的是社区协会、非营利组织和临时工作组。第二组行为者由具有不同数字实践的群体组成，他们边缘化的特性影响了数字系统的表达和追求方式。这些行为者策略性地在不同的间隙之间游走以产生特定类型的政治影响，并且经常通过他们的缺席影响智慧城市，从而对智慧城市的政治地理发生深远的作用。

## Introduction

The smart city urban planning paradigm is now both entrenched on a global level, and is deeply contested by academic scholars and community activists. A critical research agenda has developed around the smart city’s assumptions, implications and socio-political relations, with a strong focus on the new forms of governance it signals (Ruhlandt, 2018). Urban studies scholars have long understood the ways digital technologies shift cities’ forms and processes (Boyer, 1996; Graham and Marvin, 1996; Mitchell, 1996), and yet the latest wave of digital urban geographies manifesting in smart cities has raised pressing new questions stemming from new forms of data collection, data processing and technological determinism. Among these questions are who the smart city is for, who enacts smartness, and who has the right to the smart city (Kitchin et al., 2019). Both the academic literature on smart cities and the empirical phenomenon of smart cities have always been fraught and conflictual (Greenfield, 2013; Murakami-Wood, 2021; Wylie, 2018), but some common threads cast light on how it has been theorised over time.

Early critical research on smart cities tended to assume an orientation in which municipalities, regional governments and the nation-state enact programmes to embed Information & Communication Technologies (ICT) in the urban landscape. This conception of smart cities prioritises the state’s role as an active agent, with less attention paid to those subjects of state power. Research within this area noted the ways in which smart cities’ programmes were mere handmaidens to deepening capitalist processes led by coalitions of private corporations and various state scales (Greenfield, 2013; Hollands, 2015; Söderström et al., 2014). Insofar as private companies in this way frame the smart urbanisation process, they likewise serve as governmentalising institutions (Klauser and Söderström, 2016).^1^ This early formulation, which many have called the ‘top-down’ view of smart cities, frames the dominant actors as enacting their programmes on target populations, with little meaningful consideration paid to civic participation or communities’ needs.

Because of the way in which this critique circulated among critical scholars, the natural response has been to seek social justice either by contesting these top-down exercises of (digital) power or by reconceptualising the smart city ‘from below’. The latter implies seeking citizen-driven strategies situated near the top of Arnstein’s ladder of participation, where citizens hold more direct control over the deployment of smart city initiatives (Cardullo and Kitchin, 2019). Scholars have characterised this approach as ‘bottom-up’, referring to the grassroots analogy that drives such thinking (Ratti and Townsend, 2011).

A growing chorus of voices recognises the limitations of this dichotomous view of smart city politics. In this paper, we join this chorus by developing the concept of interstitiality, to understand two complementary socio-political processes related to the smart city. First, we concur with recent scholarship showing that the dichotomy of top-down and bottom-up overlooks actors influencing the ways in which the smart city manifests, but that are neither at the ‘top’ nor the ‘bottom’ of the power relation. We call these actors *interstitial* to stress their role as (powerful) mediators of smart cities, in which multiple publics coalesce in institutions such as community associations and non-profit organisations. Second, we argue that smart city visions are largely compelled by social imaginaries of those who are *interstitial to* the smart city. In a sense, this is a re-working of the idea of the digital divide; however, instead of focusing attention on those involuntarily marginalised from digital access or digital skills, these interstitial actors are construed here as those sitting uneasily within the deepening digital milieu. Together, interstitial actors impact urban political geographies by moving between various ‘scales’ of interstitiality, in order to enact particular kinds of influence that were unavailable to them at other scales. Our goal here is not to establish that these interstitial scales *exist*– we contend that such evidence is already in the literature – but instead to provide empirical grounding for rethinking interstitiality in these terms.

Conceptualising the interstices of smart cities in this way has immediate scholarly stakes as well as more long-term political stakes. First, we contend that smart cities require deeper conceptual clarity about the complex roles that various actors play, including the ways in which they shift between roles to affect political change, and that this clarity would ascribe greater agency to collectives poised to advance socially just cities. As Dowling et al. (2021) have pointed out, smart cities are *made*, and research should illuminate how they are made and by whom. Second, reconceptualising smart cities in this way involves thinking about what work smart city programmes, and digital technologies more broadly, are able to perform – and who gets to claim ownership and stewardship over this work. This line of inquiry has immense potential for a politics of justice in the context of smartness, beyond its scholarly value. Theorising both *who* the interstitial actors *are*, as well as how they perform diverse roles to impact urban political geographies, aims to nuance understanding of how smartness is envisioned, implemented and contested.^2^ Taken together, McFarlane and Söderström (2017) argue, these two sets of considerations are a key way in which smart cities research can (re-)orient itself towards creating more socially just cities.

## Situating smart urban interstitiality

At the time of writing, the smart city continued to elude straightforward definition, particularly when considering its relation to parallel urban development paradigms. Definitions abound in number, diversity of purposes and even the degree of focus on digital technologies (Albino et al., 2015). Indeed, the urban planning paradigm of ‘smart’ developed its meaning alongside a plethora of related terms such as sustainable, eco-city, resilient and knowledge cities (de Jong et al., 2015). These, and the many other articles that seek to *critically* theorise smart cities (e.g. Caragliu et al., 2011; Kitchin et al., 2019; Luque-Ayala and Marvin, 2015), are connected primarily by understanding smartness as related to material digital technologies, but often in discursive, political-economic and governmentalising ways (Hollands, 2015; Jirón et al., 2021; Marvin and Luque-Ayala, 2017; Söderström et al., 2014). In this article we largely follow Kitchin et al. (2015) in understanding smart cities as both the urban planning and administration project of embedding digital technologies into the urban fabric, and a reconfiguration of digital-urban economies.

Within this broad set of research, scholars have commonly approached the smart city as the unrolling of digital-technology programmes from the state or private corporations to the public. We denote this the *top-down* approach to capture the emphasis on various scales of the state – such as municipal government, provincial or regional government or the nation-state – and large private corporations such as IBM, Cisco, Socrata and Palantir (for examples of the former, see Calzada, 2017; Dierwechter et al., 2017; and for the latter, see Sadowski, 2020; Wiig, 2015). In other words, smartness is seen as a programme dominated and unrolled by powerful actors. Importantly, ‘top-down’ signals researchers’ focus and theoretical orientation when theorising the smart city, and is often implicit in their engagement rather than what they explicitly seek to establish. Much research within this area, then, subtly reinforces such an idea in nevertheless rich case studies on socio-technical practices as broad as policing (Jefferson, 2018) and redlining (Safransky, 2020), capital accumulation (Hollands, 2015) and firm strategies (McNeill, 2015), epistemology (Barns, 2016; Kitchin et al., 2017) and governmentality (Gabrys, 2014; Vanolo, 2014), to state only a few examples.

Sensing that the top-down focus of smart cities research neither adequately represents the initiatives’ complexity, nor accounts for socio-political resistance through individual place-making, in recent years research has responded to the top-down approach by turning attention to *bottom-up* smartness. Research tends to use this term loosely, without perfect clarity regarding what precisely constitutes ‘bottom-up’, but such literature generally emphasises individual city residents, citizens, ‘hackers’ and other persons either resisting how their smart city unfolds (Kaika, 2017), or constructing their own version of it (Irani, 2015; Perng, 2019; Schrock, 2016). This research often focuses on cities such as Barcelona, where residents hold a remarkable degree of influence over how the smart city develops (Charnock et al., 2021; Lynch, 2020), but Townsend (2013) and Picon (2015) associate the bottom-up with loosely-organised, horizontalist, often ad-hoc groups of *individuals* such as in hackathons or mapathons. As well, due to its concern with the relationship between individuals and socio-technical systems, bottom-up research often orients around issues of citizenship and subjectivity (Burns and Andrucki, 2021; Datta, 2018; Shelton and Lodato, 2019). For example, Shelton and Lodato (2019: 48) focus their analysis on ‘the voices of “average” citizens in decision-making processes’. Datta (2016) holds that top-down approaches to smartness necessitate an empirical counter-movement, which by extension results in a need for conceptual adjustment:

top-down visions of smart urbanism produce new forms of dispossession and citizenship struggles around law and legality. Simultaneously it also shows that while the trope of the smart city might be new in India, the struggle for citizenship rights and social justice at the grassroots is certainly not a new one. (Datta, 2016: 65)

Insofar as the smart cities literature focuses on these loose amalgamations of individuals when theorising the bottom-up, it departs from more classic formulations such as the literature on urban social movements and activist networks as captured in the metaphor of ‘the grassroots’ (e.g. Castells, 1983; Routledge, 2003).

Couched within notions of an ‘alternative’ smart city (Söderström et al., 2014), scholars often see broad potential for more socially just cities here; Greenfield (2012: n.p.), for instance, envisions a ‘technical [system] in which value is both produced from the bottom up and primarily returned to the parties responsible for its production’. Even Shelton and Lodato (2019), who question the current political purchase of the ‘smart citizen’ discourse because of the way it (in practice) forgoes meaningful participation in favour of lip service, imply in this critique that an *actual* engagement with smart citizens would lead to more just cities.^3^

Despite the important conceptual contributions of both top-down and bottom-up visions of smart cities, scholars are increasingly expressing discomfort with this overly reductive ordering (Chang and Chung, forthcoming). Luque-Ayala and Marvin (2015) cast early suspicions on the simplicity of this dichotomy, drawing attention to

other forms of [smart urbanism (SU)] being rolled out through a multiplicity of dispersed and disconnected initiatives under the leadership of communities, ad hoc volunteer groups and local organisations. … [C]ommunity involvement in SU shows that notions of ‘top-down’ and ‘bottom-up’ do not adequately reflect the complexity of issues at play. (Luque-Ayala and Marvin, 2015: 2113)

Likewise, Cowley and Caprotti (2019) argue that smart cities reject the social ordering and epistemological dominance of planning regimes (the top-down ontology), but instead of a purely ‘bottom-up’ collection of active individuals, smart cities prioritise the participation of a range of non-profit and digital innovation organisations. Further, discussing the South African civil society organisation Social Justice Coalition Cape Town, Odendaal (2016: 82) argues that such organisations ‘… do represent a “smart city from the bottom up”, but mainly as a challenge to city discourses and, more importantly, as a monitoring strategy’. An important distinction arises here: while much of the bottom-up literature focuses on governable individual citizens, Odendaal emphasises a different scale, where a coalition of 11 branches and 40 partner organisations advocate *on behalf of* what others conceive as the ‘bottom’ in bottom-up. We contend that this distinction is an important one that requires conceptual attention.

This discomfort with the bottom-up/top-down dichotomy brings to mind the long tradition of urban research with community organisations and other actors that we here call ‘interstitial’. While a comprehensive review of this literature is beyond the scope of the present article, we do find it particularly useful to think through the ways that past research has shown the strong political sway wielded by community activism, organising and coordination (see, e.g., Cahill, 2007; Elwood, 2006; Heynen, 2010; Tretter, 2016). For example, calling such actors ‘intermediaries’, Moss et al. (2011: 2) argue that ‘such intermediaries play an important, but hitherto neglected, role in reshaping the relations between production and consumption relating to urban infrastructures’, which we in this article extend to smart cities. In the edited volume in which they make this argument, each chapter contends with the potentially radically transformative role that ‘intermediaries’ can play on urban infrastructures. Further, in their work on community and diverse economies, Gibson-Graham (2006) also brought to our attention that our epistemological framings serve a performative function: by theorising capitalism as ‘the hegemonic, or even the only, present form of economy’ (p. 2), scholars miss an impressive breadth of present economic activity that underwrites urban processes. We translate this lesson to smart cities as saying that in theorising bottom-up or top-down smart cities, we necessarily overlook the ‘middle’ actors that urban geographers have long established as impacting urban geographies.

## Methodology

In the following sections, we draw from a five-year ongoing qualitative study examining smart city discourses in the context of Calgary, Alberta. For this project we combine the extended case method (Burawoy, 1998), a theory-driven abductive approach helpful for deriving multi-scalar theoretical propositions from empirical observation of small-scale processes, and the database ethnography (Burns and Wark, 2020), a framework for understanding the production of social meaning within digital ecosystems. Calgary is a fruitful site to explore these questions as a city deliberately positioning itself as ‘smart’, which prides itself on stakeholder engagement in municipal decision-making, and which was in 2006 home to the highest number of non-government organisations per capita in Canada (CCVO, 2010).

Our specific methods include participant observation, semi-structured interviews, archival analysis of reports and news sources, and ethnographic notetaking. As participant-observers, we have attended nearly 30 events organised by both local and national actors ranging from community association meetings, regional special interest groups (e.g. organised on Meetup.com and Facebook), municipal public engagement sessions and city council meetings. Across all these activities we have identified key actors and decision-makers involved with envisioning and enacting urban politics with or regarding digital technologies, and with them have conducted 31 in-depth semi-structured interviews (Rubin and Rubin, 2005). We triangulate these methods with reflexive ethnographic note-taking (Emerson et al., 2011) to texture our analysis and situate each researcher’s account of events, processes, and actors within the smart city discourse.

Our analytical framework aligns with discourse analysis, a common approach in qualitative research that allows the researcher to contextualise the social and historical circumstances within which knowledges are produced, articulated and given meaning (Dittmer, 2010; Jørgensen and Phillips, 2002). We pursued our analysis bearing three parallel objectives. First, we aimed to illuminate the range of actors, institutions and coalitions that are neither the government, nor corporations, nor loosely coordinated individuals – those which operate in the ‘interstices’ of the smart city. Second, we identified the various processes, relationships and apparatuses utilised by both interstitial actors and smart city administrators to produce influence. Third, we sought to situate these actors and processes within the uneven geographies of the digital divide. In pursuing these objectives, we illuminate these often overlooked interstitial actors and illustrate the significant roles they can play to influence manifestations of urban smartness, and urban politics more broadly. Below, we proceed with our analysis by first describing those actors in between the top-down and bottom-up; we then conceptualise those who are on the margins of the smart city; and finally we theorise the way interstitial actors often move between the interstices as a politically powerful strategy of influencing the smart city.

## *Inside* interstitial actors

Calgary’s smart city initiatives, while of course interfacing both top-down and bottom-up efforts, more prominently emerge from the work of actors identified in the broader literature that we characterise as *interstitial*. They are neither properly top-down nor bottom-up but instead exist in two forms of interstices. The first is a collection of civil organisations such as community associations, non-profit organisations, charities and community association task groups. These actors are interstitial in the vertical organisation of power relations between the state/corporations and individual citizens. We discuss the second form of interstitiality in the next section. We show some partial and incomplete, but illustrative and pseudonymised (when necessary), examples of each of these sorts of actors in Table 1. This table is neither exhaustive nor definitive, but rather a heuristic illustrating some of the key actors that emerged in this study. The value of this heuristic is in highlighting the relationality in which some actors operate. In what follows, we show who these actors are within Calgary, in order to show how they move between multiple positionalities to enact particular forms of political influence on the urban, via smartness.

**Table 1.** Interstitiality can be conceived along two axes – those inside the smart city and those outside of it. Each group of interstitial actors exerts different influence on the smart city.

	Example groups	Forms of influence
Inside interstitial actors		
Non-profit organisations	Cybera, CivicTechYYC	‘Smart City Community Team’, frequently consulted by City planners
Community associations	Hillhurst-SunnysideCommunity Association	Legally incorporated entities that have strong representation in formal urban planning meetings; proactively produce data and digital representations of their communities
Outside interstitial actors		
Newcomers	People who haverecently immigrated	Different engagement with ‘smart’ digital technologies; different relationship with digital literacy and inclusion
Age demographics	Youth either prior to firsttechnologies, or those who use technologiesdifferently; the elderly	

### Delineating the inside interstitial actors

In between the municipal, provincial and national governance bodies on the one hand, and urban residents’ activism on the other hand, lie a range of civil organisations that hold a great deal of power in envisioning and enacting the Calgary smart city. The number of such organisations in the province of Alberta should not be understated: as mentioned above, Alberta has the highest per-capita number of non-profit organisations in the country (CCVO, 2010). Most prominent here are non-profit organisations like Cybera, a ‘digital accelerator responsible for driving the province’s economic growth through the use of digital technology’,^4^ and CivicTechYYC, a ‘community based group that is part of a global movement to leverage technology for public good’.^5^ As one Cybera administrator, Karen, described to us in an interview, her organisation mission has expanded its scope from digital networks access to other aspects of digital human engagements key to the smart city. Karen explained that her organisation looks:

to enable computational thinking and improve digital literacy across the province … and how that enables people to be innovative with technology. And understand how to implement it and the disruption that it creates so we can all participate in the digital economy.

They do this in collaboration with a variety of actors including primary schools, universities, government institutions, non-profits, start-ups, First Nations communities and agriculturalists.

In contrast, CivicTechYYC pursues a different strategy by creating collaboration spaces where multi-sector volunteers come together for hackathons and monthly collaboration meetings. The CivicTechYYC community sees itself as a ‘connector within Calgary and the various parts of the [technology] ecosystem’ while also facilitating the development of technology-based projects ‘to make Calgary an even better place to live’.^6^ Many other such non-profit organisations, and charity organisations like the Calgary Homeless Foundation and the homelessness and poverty reduction shelter The Mustard Seed, are involved in producing and analysing data and digital technologies in order to contribute to Calgary’s smart city programme as a whole.

Calgary’s unique political-geographic history has led to an outsized role for community associations in municipal politics (Davies and Townshend, 1994). More than mere channels of community members’ political energies, community associations materially frame debates, promote re/development and influence city programmes. Our interviewee Rowan, the Planning Director for a community association, informed us that ‘from a planning perspective it’s really interesting because [community associations] could appeal anything that happens in their area because they’re considered legally an affected party’. Rowan described how a neighbouring association developed a digital system for automatically appealing any proposed developments in their district, therefore securing a voice with the city council each month at review.

### Influence on the smart city

More than mere conduits relaying the needs, knowledges and desires of their constituents, interstitial actors play a key role in framing how the smart city unfolds. This is an influence on both its material dimensions of physical sensors and circulation of capital, and the symbolic-discursive dimensions of how the smart city is conceived and the way its value is communicated. They wield this influence by intervening in formal city planning processes, and by enacting, on their own terms, data collection and representation practices. Formal city staff actively cultivate these interstitial energies and direct them into the smart city plan. Our interviewee Jean, a leader on the city’s smart city programme, told us that Calgary is ‘taking [the term “smart city”] back from industry – we’re starting to learn that platforms will not just solve all our problems. Don’t send us your vendors and platforms; instead, show us you’ve spoken with our communities.’ For Jean, Calgary is pioneering a new smart paradigm in which urban challenges are represented not by the city government or private businesses – each of which preoccupied early scholarly smart cities literature – but by groups of individuals. By accentuating ‘communities’ rather than ‘citizens’ or ‘residents’, Jean emphasises the collective nature of those the city government prioritises.

In Calgary, non-government organisations and groups, formal and informal, are sought out as key collaborators in smart development. To engage these actors the city has established formal participation protocols as well as more experimental knowledge production activities with Calgarians. Our interviewee Zoe leads an important resilience programme for the city that has co-articulated in complex ways with Calgary’s smart city work.^7^ In developing components of their resilience strategy, Zoe and her colleagues widely recruited participants and organised collaboration sessions with representatives from a diversity of businesses, organisations and communities. As Zoe described to us, ‘We had the health region, the education system, we had not-for-profits, banking, finance … faith groups. I mean, I can’t even explain to you how many… But yeah, it was very much about organizations of influence but also equity seeking communities.’ Using these frameworks of participation, interstitial actors are provided formal channels to articulate their unique knowledges and visions into official municipal strategies.

Indeed, the city has explicitly delegated some of its smart city work over to these interstitial actors. In its submission for the 2018 Canada-wide Smart Cities Challenge, its 16 explicitly listed key partners are what we are calling interstitial organisations representing a broad spectrum of mandates, from Cybera to the Calgary Homeless Foundation (City of Calgary, 2018, 32). This sort of delegation of smart urban governance to interstitial actors occurs in many other similar contexts.

However, interstitial actors do not merely passively wait for the city’s consultation or delegation. Many community organisations themselves have enacted data collection and representation practices to contribute data more important to – or reflective of – the community association and its members. Our interviewee Tanya, who was at one time the president of her local community association, launched a programme to collect data about parks usage in her community. With a small staff, they recorded such information as which park fixtures are used by patrons, how long patrons spent on each fixture, and so on. They have mobilised these data in multiple ways: they have been able to better target community health programmes, petition city departments for park equipment upgrades, and have stronger control over how their community enters smart cities discourses. Tanya successfully captured the city’s attention with these efforts, at one point taking a leadership position in a city department related to parks.

## *Marginal* interstitial actors

While the first form of interstitiality focuses on the vertical scales between top-down and bottom-up, the second form of interstitiality is the set of actors on the margins of smartness whose spectre compels digital urban programmes.^8^ This is a horizontal interstitiality, in that it is still neither top-down nor bottom-up, but instead situates actors strategically outside (though never entirely *outside*) the discursive and material territory of smart urban practices, as we show below. This group is comprised of a plethora of actors traditionally conceived as on the ‘lacking’ side of the digital divide: most prominent in Calgary are immigrants, the elderly and youth. However, we contend that the digital divide conceptual framing does not precisely capture this collection of actors. Whereas the digital divide would construe these actors as involuntarily ‘left behind’ in an increasingly digital society (Gilbert, 2010), we conceive of them as instead *interstitial* to ascribe deeper agency to their subject position, as in most cases these actors have been socialised to either use digital technologies *differently, not yet* use them, or voluntarily abstain altogether. These actors enact their influence on smart cities from its *interstices*– its figurative margins. In this way, they function as a spectre: fear of these interstices compels smart city programmes to take some actions and development plans over others, in order to draw in these people from the margins.

In Calgary, this form is most clearly evidenced in two categories. The first is comprised of poor people, immigrants, youth, and the elderly.^9^ For our interviewee Granger, a city employee playing a leading role in Calgary’s smart city programmes, Calgary’s approach is characterised by its focus on digital *literacy* rather than simply access to digital technologies; that is to say, the city understands that expanding access to technology presupposes that target populations are able to effectively leverage and engage those technologies. In response to our question about his data needs, Granger told us:

[I’d like] better information on what people’s barriers are – data-wise… where people are getting online and where they’re not, and why they’re not. So what are those barriers? Is it literacy? Is it digital literacy? Is it affordability, is it lack of infrastructure? Data from the internet providers would be great.

In this quote, Granger gestures towards the way in which those people facing ‘barriers’ remain in his consciousness as he works within socio-technical systems like smart cities. He acknowledges his lack of empirical knowledge of the sources of what he elsewhere calls ‘digital disparities’, but most importantly, his awareness of these barriers and disparities consciously figures into how he strategises for smart city programmes. His imaginary of interstitial actors, while hazy due to a lack of data, remains an important factor in his work.

These interstitial actors can also influence the first form of interstitial actor, further complicating the utility of thinking in top-down and bottom-up terms. In Calgary, community associations, non-profit organisations, charities and ad-hoc task groups often invoke this second group of actors when advocating particular smart programmes and digital practices. Shier, who is president of a community association representing predominantly poor and immigrant communities, pointed in particular to the populous South Sudanese community in her neighbourhood:

You know, they never pay on debit, things of that nature, whereas most, I suppose European Canadians, we’re pretty used to our credit cards and our debit cards. … And then, I don’t know if this is just generational or if it’s also community-based as well, but just the types of communication. They just really don’t want to talk over email, they want to talk to you in person, or they want to talk to you on the phone.

Here, Shier characterises the ways in which this community’s digital practices diverge from practices common to the assumed subjects of the smart city, who are usually framed as tech-savvy and saturate their daily lives with digital interactions (Burns and Andrucki, 2021). While they of course still use digital technologies, this use is most often not easily incorporated into popular conceptions of the smart city. Indeed, one might argue that these practices sit askew to the datafication imperative as well (Sadowski, 2020). This conflicts with Shier’s attempts to digitise operations at the community association; her primary employment is with a popular digital payments company, and she has transferred all community association finances to that company’s point of sale platform. Even as she pursues increasing digitalisation of her community association’s work, the spectre of interstitial actors reminds her of digitalisation limits.

The second category of actors within this form of interstitiality is comprised of organisations and institutions without digital capacity sufficient to collect, manage, analyse and visualise data streams construed as part of the smart city. In most instances these should not be understood as experiencing marginalisation equivalent to the other category, but are still ill-equipped to be active, physically present actors within smart city programmes. Here, we have in mind organisations like the Calgary Women’s Emergency Shelter and the Calgary Homeless Foundation, which have both been assisted by the loosely-organised group Data for Good – Calgary. These two charities exemplify a common story: they have large datasets generated from their daily operations – calls, visits, fundraising, financing, etc. – and no capacity to analyse the data. Insofar as the smart city presumes its denizens will produce, analyse and act on *data*, these organisations are situated within its interstices. In our multiple interviews and participant-observation with Data for Good – Calgary, one of its prominent members, Alex, has regularly referred to the ‘data ecosystem’ that characterises the province-wide drive for smartness. In short, Alex sees this organisation as a core component of Calgary’s smart city and the province of Alberta’s constellation of smart programmes.

In these activities, organisations lacking a significant data analysis component are interstitial: while they are not actively promoting one vision or another of the smart city, their inability to ‘fully’ contribute haunts actors who drive the smart city’s development. The absent-presence of these actors means that the larger smart city context assumes particular forms to accommodate or to absorb these interstitial actors. That is to say, it manifests differently in response to interstitial actors’ presence in the city.

Laclau and Mouffe’s (1985) notion of the ‘constitutive outside’ helps us to make sense of this absent-presence.^10^ As Laclau and Mouffe have shown, a phenomenon such as the smart city *depends on* that which its proponents claim is its ‘outside’ for its own identity formation and symbolic relations (see also Mouffe, 2000). The smart city must contrast with non-smartness in order for it to be understood as a coherent concept. For Laclau and Mouffe (1985), the constitutive outside is rooted in a political antagonism, between insider and outsider, friend and enemy, community and other; the point is not necessarily that this relationship is hostile, but that its quality is marked by such a process of demarcation and signification. The smart city’s interstitial actors in many ways can be understood as its constitutive outside: they mark the discursive limits of a digital city that claims to be participatory, efficient and democratic, because they are illegible within smart socio-technical systems (Mouton and Burns, 2021). This means that, although we argue that they exist in the interstices of the smart city, they are never truly *outside* of it, and in fact are a core component of what qualifies the smart city. To say that they are in the interstices, then, is to say that they are not the ones ‘steering the ship’, but that their existence still shapes the way in which the smart city unfolds.

## Mobility in the interstices

Above, we alluded to the ways that actors strategically negotiate different relationalities and positionalities within the smart city interstices, in order to influence urban politics of smartness. Many of our interviewees take on, create new, or switch between interstitial roles, organisations and spaces to engage other actors and generate support for particular smart city initiatives. For example, an actor may simultaneously serve as a non-profit organisation administrator, a community association board member, and spouse to a city counsellor – as was the case for one of our research subjects. This enabled the person to strategically mobilise their multiple positionalities with profound consequence for their interstitial actor organisations. Or, like another research subject, one may serve as a municipal technology worker, university instructor and CivicTechYYC member. In this case, the actor moved *into* the interstices and *between* groups to mobilise support and leverage their initiatives from different angles within the smart city hierarchy.

Across multiple interviews, actors in the second form of the interstices would work to rearticulate what ‘smart’ means in order to redraw the discursive bounds of the smart city and its interstices. These actors exemplified an additional form of mobility: one in which concepts, terms, and articulation are made flexible in order to strategically enter into smart city dialogues. Actors would, for example, draw attention to failed smart city programmes, or programmes that, despite working as intended, resulted in unexpected outcomes or in marginalised populations reflecting with some variant of the phrase ‘That doesn’t seem very smart to me.’ In doing so, they highlight their own marginality to smart discourses, and redraw those boundaries in order to claim a position in *naming* smartness.

This mobility has important ramifications for thinking about the agency that each role has in a smart socio-technical system (Moss et al., 2011). We see here that interstitial actors are neither passively acted upon, nor static in their positionalities. Instead, actors strategically negotiate multiple roles and relations in order to intervene in how the smart city unfolds. At the same time, the discursive relations of the terms, concepts and articulations of smartness are also fluid, and are often redrawn in order to achieve similar outcomes. This conceptualisation paints a more complex picture than the top-down and bottom-up views of smart cities, and sheds additional light on the emerging conversations regarding the gaps in those views.

## Conclusion

In this article we have developed the concept of interstitiality for smart cities, in order to contribute deeper understanding of the actors that smart cities scholars increasingly recognise as under-theorised and yet important to smart cities’ development. Here, we have focused on two broad sets of interstitial actors: those between top-down and bottom-up smartness, and those on the *interstices* of the smart city. The former, most notably comprised of community associations, non-profit organisations, ad-hoc civil society working groups and charities, are often formally integrated into the planning process and actively contest the ways in which the smart city represents their communities. The latter, what we characterise as a spectre of those who have not smoothly assimilated into the smart city and who are often seen as *not yet* properly engaging digital technologies, are often comprised of youth, recent immigrants, the elderly and others on the ‘wrong’ side of the digital divide. Despite being physically relatively absent from the formal planning process, their presence is felt as a need for deeper integration into digital socio-technical systems. We contend that these actors’ political sway comes from their ability to move between different scales and discursive bounds in order to strategically influence the smart city.

Dedicating some focus to these groups accomplishes three important tasks. First, our contribution advances a theorisation of the complex politics underwriting digital technologies and the urban. We argue that interstitial actors are neither passive recipients nor mere conduits for smart policies, but that current epistemological framings of the smart city have historically relegated them as such. Rather, interstitial actors actively shape smart city programmes even when physically absent from the arenas of formal planning. Here we provide a conceptual framework to help advance the increasing discontent with top-down, bottom-up framings of smart city politics; we do not seek to establish the factuality of interstitial actors, because many others do this, but we may deepen the theorisation of those politics by thinking them through the framework of interstitiality. We thus speak to the question raised by Moss et al. (2011: 8): ‘How far and in what ways are intermediaries transformative to urban infrastructure systems?’

Second, our argument opens new avenues for activists, policymakers and interstitial actors to intervene in smart city politics, towards more just cities. In this article we have aimed to advance the form of social justice advocated by Blue et al. (2019) that seeks to work towards parity of participation, and Mackinnon et al. (forthcoming) who seek a multi-directional, contextually contingent notion of justice around smartness. If smartness is framed, executed and contested not just from the top-down and the bottom-up, but in powerful ways from its interstices, then perhaps such actors could be mobilised for meaningfully envisioning a more just smart city – or figuring out what’s ‘next’ after smartness. Our contribution can be read as North America-centric, as the meaning and power of interstitial actors like community associations are very different in other regions of the world; however, we maintain that our primary contribution here is in thinking more deeply about the relationality and influence of complex sets of actors that are neither the top-down nor the bottom-up, and in this way our insights may still hold value as they travel elsewhere. It is also flexible, able to incorporate or taxonomise actors that we do not list here: perhaps in some places small and medium enterprises should be considered top-down because of a particular spatial relation of power, but in other places they could be considered interstitial, for instance.

Third, our research builds on urban studies’ longstanding interest in how organised groups of individuals co-produce and resist urban processes. While this work has traditionally looked at urban social movements (e.g. Routledge, 1997; Ward et al., 2018), we focus here on a technologically-saturated form of urbanism that enrols actors into different sets of roles and relationships.

While smart cities are often thought to be handmaidens for private capital accumulation, sometimes occurring through the neoliberalisation of municipal government’s roles and responsibilities (Greenfield, 2018; e.g. Hollands, 2015), here we paint a more complex picture. By re-emphasising the role of interstitial actors not *just* as subjects enacted upon, nor merely an amassing of individuals, but instead as active collective agents of smart urban change, we contend that the smart city produces fertile ground for new political-economic relationships, such as the ‘non-profit industrial complex’ (Rodriguez, 2007), in which capitalist urban logics percolate through spaces and institutions purportedly outside of capitalist relations. We hope that by synthesising smart cities research in these ways, and by re-emphasising the agency of interstitial actors, we will be better able to observe and understand the complex political geographies of smart cities.

## Notes

1. That is to say that they produce governmentalising frames of reference, not that they are government institutions.

2. Moss et al. (2011) argue that this is a pressing research question emerging around urban energy infrastructures, and we contend that it applies to smart cities as well.

3. We do not disagree with this assessment; we point it out simply to link these debates with other debates on social justice in the smart city.

4. From Cybera’s homepage description, accessible here: https://www.cybera.ca/.

5. From https://civictechyyc.ca/.

6. From a public CivicTechYYC talk, available at: https://www.youtube.com/watch?v=lNrn_6f0yuc.

7. This is not unusual, as urban resilience has across many cases been identified as coupled with smart city programmes (Kaika, 2017; Leitner et al., 2018) or enfolded in more ‘chameleonic’ ways (Caprotti and Cowley, 2019).

8. While the term ‘spectre’ is often associated with Derrida’s (1994)*hauntological* read of Marx and Engels (1848), we use it in the more general sense of a foreboding spirit that is simultaneously present and absent, compelling particular actions out of apprehension or fear.

9. We combine these into a single category because, for analytical purposes, they function in similar ways in smart city actors’ imaginaries.

10. To be sure, while our argument presumes the physical absence of these interstitial actors from the formal planning process, there may be empirical cases in which they, as a collective, have actually intervened in them. We do not know of a study accounting for such interventions.
